# Understanding, experience and attitudes towards artificial intelligence technologies for clinical decision support in hearing health: a mixed-methods survey of healthcare professionals in the UK

**DOI:** 10.1017/S0022215124000550

**Published:** 2024-09

**Authors:** Babatunde Oremule, Gabrielle H Saunders, Karolina Kluk, Alexander d'Elia, Iain A Bruce

**Affiliations:** 1 Paediatric ENT Department, Royal Manchester Children's Hospital, Manchester University NHS Foundation Trust, Manchester, UK; 2 Division of Infection, Immunity and Respiratory Medicine, School of Biological Sciences, Faculty of Biology, Medicine and Health, University of Manchester, Manchester, UK; 3 Manchester Centre for Audiology and Deafness, School of Health Sciences I Faculty of Biology, Medicine and Health, University of Manchester, Manchester, UK; 4 Department of Public Health, Policy and Systems, University of Liverpool, Liverpool, UK

**Keywords:** Artificial intelligence, attitude of health personnel, audiologists, general practitioners, otolaryngologists, United Kingdom

## Abstract

**Objectives:**

Clinician acceptance influences technology adoption, but UK health professionals' attitudes towards artificial intelligence (AI) in hearing healthcare are unclear. This study aimed to address this knowledge gap.

**Methods:**

An online survey, based on the Checklist for Reporting Results of Internet E-Surveys, was distributed to audiologists, ENT specialists and general practitioners. The survey collected quantitative and qualitative data on demographics and attitudes to AI in hearing healthcare.

**Results:**

Ninety-three participants (mean age 39 years, 56 per cent female) from three professional groups (21 audiologists, 24 ENT specialists and 48 general practitioners) responded. They acknowledged AI's benefits, emphasised the importance of the clinician–patient relationship, and stressed the need for proper training and ethical considerations to ensure successful AI integration in hearing healthcare.

**Conclusion:**

This study provides valuable insights into UK healthcare professionals' attitudes towards AI in hearing health and highlights the need for further research to address specific concerns and uncertainties surrounding AI integration in hearing healthcare.

## Introduction

The global shortage of hearing health professionals and the increasing burden of hearing loss and ear pathology are well documented.^1,2^ Artificial intelligence (AI) technologies have gained significant attention in healthcare for their potential applications in clinical decision support, and helping to deal with significant increases in global healthcare need.

In the field of hearing health, AI holds promise for improving diagnostic accuracy, treatment planning and patient management to help combat the global shortage of healthcare workers and increase the efficiency of healthcare delivery.^3–6^ Examples include the use of a dermatology AI diagnostic tool to triage skin cancer referrals, resulting in 799 out of 2023 (37 per cent) patients being discharged without needing to attend a hospital appointment during a 6-month pilot, and a radiology AI tool supporting radiologists reporting magnetic resonance imaging scans, which resulted in a significant reduction in reporting time and superior or equivalent inter-observer agreement.^7,8^

The use of AI technologies in healthcare raises ethical issues around patient safety, empathy and/or patient-centred care, data security, professional de-skilling, and apportioning blame and/or fault, which can affect attitudes and the acceptability of AI technologies.^9–14^ While previous studies have explored AI in healthcare in general,^14–17^ limited research has focused specifically on the attitudes of healthcare professionals towards AI technologies in hearing health.

Healthcare professionals in AI-advanced medical specialties, such as dermatology and radiology, generally have positive attitudes towards the integration of AI technologies into clinical workflows. However, a minority of professionals expressed concerns about potential job displacement in the future.^18^ Practicing doctors were more inclined to believe that certain aspects of their job roles might be replaced by AI technologies, while students were more inclined to believe that entire job roles could be lost.^19^ Understanding healthcare professionals' perspectives on AI in hearing health is crucial for the successful integration and adoption of these technologies.

This study aims to fill this gap by examining the understanding, experience and attitudes of healthcare professionals in the UK towards AI technologies for clinical decision support in hearing health. Understanding these important stakeholder's attitudes will support user-centred AI development and increase the likelihood that AI technologies in hearing health will be introduced into clinical practice.

The objectives of this study were (1) to assess healthcare professionals' understanding of AI technologies in hearing health; (2) to examine healthcare professionals' experience and usage of AI in their practice; and (3) to explore the attitudes and opinions of healthcare professionals towards AI, including concerns, perceived benefits and potential challenges.

This research will provide insights into the current state of AI adoption in hearing health among healthcare professionals in the UK. The findings will contribute to a deeper understanding of the attitudes and concerns of healthcare professionals, guiding the implementation of AI technologies in hearing health. The outcomes will enable policymakers and healthcare organisations to address barriers to adoption and facilitate the effective integration of AI into clinical practice.

## Materials and methods

### Literature review

A scoping review of the literature for English language publications related to attitudes to artificial intelligence for healthcare applications amongst healthcare professionals was performed. The Medline (PubMed), Google Scholar and Cochrane Library databases were searched using relevant keywords including ‘artificial intelligence’, ‘AI’, ‘machine learning’, ‘healthcare professionals’, ‘attitudes’, ‘opinions’ and ‘acceptance’. The included studies were those that contained quantitative and qualitative measures of healthcare professional's attitudes, opinions, concerns and views on AI technologies in healthcare. Studies that did not include these measures were excluded. In total, 316 results were retrieved, with 16 full texts included after review (Supplementary Material 1).

### Ethical considerations

Ethical approval was granted by the University of Manchester Ethics Committee (Ref: 2023-16190-27304).

### Survey design

An open online questionnaire survey method was chosen as a pragmatic way of reaching a wide audience of professionals. The design and reporting of this study adhere to the guidelines provided by the Reporting Results of Internet E-surveys framework.^20^

### Study development

The target audience for the survey were practicing general practitioners, audiologists, ENT specialists and ENT specialty doctors and associate specialists in the UK. UK-based general practitioner trainees (general practice specialty trainee year 1 and above), UK-based ENT trainees (specialty trainee year 3 and above) and doctors in non-training roles with commensurate experience were also eligible to participate. Professionals not fitting these criteria were not eligible to participate.

Articles retrieved from the literature review were synthesised to identify relevant themes and used to design a questionnaire. The questionnaire was pilot tested with 10 participants (3 ENT specialists, 3 GP trainees and 4 audiologists). Following their input, some items in the questionnaire were refined. The final version of the questionnaire can be found in Supplementary Material 2.

### Dissemination of survey

The survey was administered using the Qualtrics^TM^ online survey tool. An invitation to participate in the study, the participant information sheet and a link to the questionnaire were distributed via email to members of Health Education England Northwest, the British Association of Audiology, the British Society of Audiology and ENT UK by the professional bodies (Supplementary Material 3) and to professional contacts. The British Society of Audiology also advertised the survey on their website (https://www.thebsa.org.uk/research/). The researchers had no direct contact with survey respondents and did not collect any identifiable information.

The survey window was open between 3 March and 30 May 2023. One reminder was sent during the survey window to enhance the response rate and mitigate against non-response bias. Participation was voluntary. Responses were collected anonymously and participant internet provider addresses were not collected. No incentives were offered for participation.

Ticked checkbox responses were included at the start of the questionnaire after the participant information to capture consent. Forced responses were employed to increase the rate of fully completed questionnaires. All participants viewed the questions in the same order. Adaptive questioning (conditional display of occupation and training grade) was employed for the question on the participant's occupation to reduce the overall number of questions participants had to answer. There was one question displayed per page. A single open-ended question was included at the end of the questionnaire to allow participants to elaborate on their views on the topic of the questionnaire.

Participants were able to pause the questionnaire and return to complete it later as long as it was completed by the end of the survey window. A ‘back’ button was included to allow participants to change their responses. Respondents were prevented from making multiple submissions using cookies to assign a unique user identifier to each participant's electronic device.

### Analysis

The statistical analysis for the quantitative data was conducted using RStudio. Descriptive statistics were computed and a comparison of variables between groups (general practitioners *vs* audiologists *vs* ENT specialists) was performed using the Kruskal–Wallis test. A *p* value of less than 0.05 was considered statistically significant. Post hoc testing was not carried out as there was no statistically significant difference between the groups.

Inductive thematic analysis was used to analyse the qualitative data. The lead author (BO) read the responses multiple times to achieve familiarisation with the responses, then codes (descriptive labels) were generated to capture the key concepts and ideas. These codes were then organised into themes and the themes were subsequently analysed and interpreted by two authors (BO and A d'E).

## Results and analysis

### Respondent characteristics

One hundred healthcare professionals responded to the invitation to participate, completed the informed consent checkboxes and commenced the survey. Ninety-three participants completed the survey, giving a completion rate of 93 per cent.

The mean age of respondents was 39 years (range 22–58 years, standard deviation 9.13 years), with female participants constituting 56 per cent of the participants. The participants included 21 audiologists, 24 ENT specialists and 48 general practitioners. ENT consultants and general practitioners with more than 10 years’ experience comprised 30 per cent of their occupational groups, while audiologists had a diverse distribution of Agenda for Change grades including non-Agenda for Change bands 5 to Band 8b. ENT and general practitioner trainees each comprised 54 per cent of their occupational groups.

**Figure 1.**
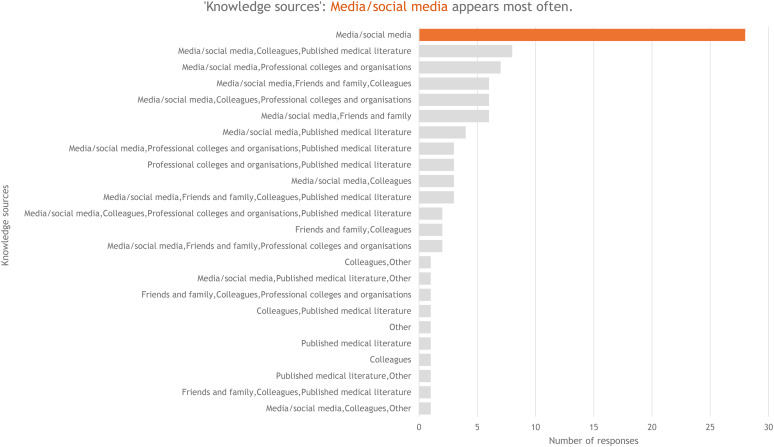
Sources of knowledge about artificial intelligence. Media and/or social media appear most often.

At baseline, participants reported a high level of confidence using everyday technology such as computers and smartphones, with 90 out of 93 participants (93 per cent) rating themselves ‘extremely confident’ or ‘somewhat confident’. The majority of the participants (90 out of 93, 97 per cent) were familiar with the concept of AI, and 80 out of 93 (86 per cent) were aware of AI applications in healthcare. Social media emerged as the dominant source of knowledge about AI, although other sources, such as science podcasts and research and development in laboratories, were also mentioned (Figure 1). Two participants (2 per cent) had received some formal training in AI in healthcare: 1 had completed a certificate-level course and the other did not elaborate.

Regarding AI usage, 14 participants (15 per cent) reported having used AI in their practice, primarily for literature searches, cochlear implant management, administrative tasks and patient triage. The remaining participants had not used AI (*n* = 73, 78 per cent) or were unsure (*n* = 6, 6.5 per cent).

Participants expressed varying opinions on AI, with the majority (69 per cent) reporting that if an AI algorithm were to have a different opinion to theirs, they would rely on their judgment rather than that of the AI algorithm. Twenty-five participants (27 per cent) were unsure about what they might do in this scenario, and 3 (3 per cent) reported that they would use the recommendation of the AI algorithm.

### Attitudes to artificial intelligence in healthcare

**Figure 2.**
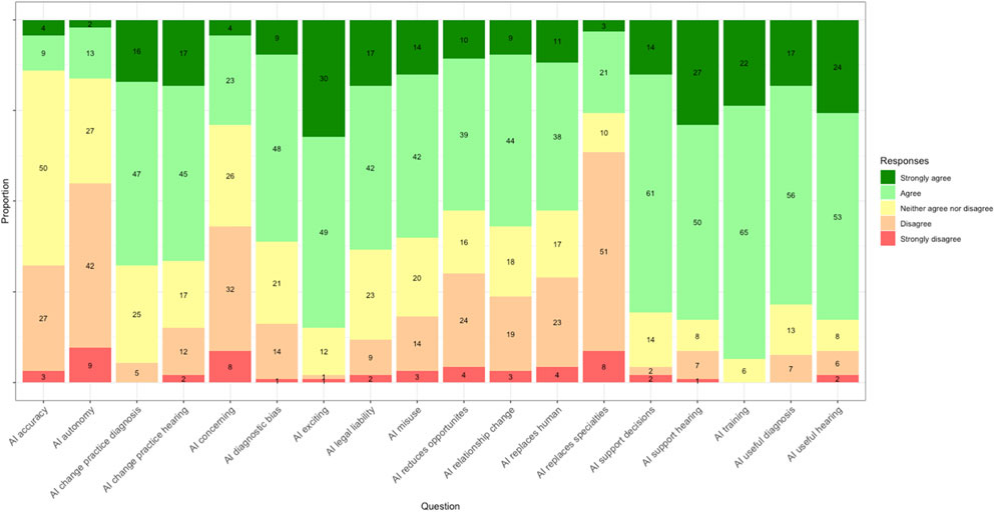
Attitudes towards AI technologies amongst hearing health professionals. See Table 1 for full question statements.

Participants' attitudes towards AI in hearing healthcare are summarised in Figure 2 and a key to the full question statements is given in Table 1. Participant responses were measured on a five-point Likert scale (1 = strongly disagree; 2 = disagree; 3 = neither agree nor disagree; 4 = agree; 5 = strongly agree). A mean score for each statement was calculated, with 1 being the minimum possible score and 5 the maximum. A higher score denoted a more positive attitude, and a lower score denoted a more negative attitude.

**Table 1.** Key to terms used in Figures 2 and 3

AI accuracy	In general AI technologies are more accurate than healthcare professionals at diagnosis
AI autonomy	AI will remove my autonomy in making medical diagnoses
AI change practice diagnosis	AI software that could help with diagnosing ear conditions would change my practice
AI change practice hearing	AI software that could help with measuring hearing thresholds would change my practice
AI concerning	New AI technologies in healthcare concern me
AI diagnostic bias	I have concerns about diagnostic bias in AI technologies in healthcare
AI exciting	New AI technologies in healthcare are exciting
AI legal liability	I have concerns about legal liability when using AI technologies in healthcare
AI misuse	I have concerns about misuse of data with AI technologies in healthcare
AI reduces opportunities	Using AI technologies in healthcare may reduce human physicians' learning opportunities and knowledge
AI relationship change	AI will change the relationship between members of my profession and their patients
AI replaces human	Some human healthcare professionals will be replaced by AI in the foreseeable future
AI replaces specialties	AI will replace some medical specialties in the near future
AI support decisions	I would use an AI software and/or program to support my clinical decision-making when diagnosing ear conditions
AI support hearing	I would use an AI software and/or program that can assist with measuring hearing thresholds if I knew the technology had good accuracy
AI training	AI in healthcare should be part of our professional education and training
AI useful diagnosis	AI software that could help with diagnosing ear conditions would be useful in my practice
AI useful hearing	AI software that could measure hearing thresholds would be useful in my practice

AI = artificial intelligence

**Figure 3.**
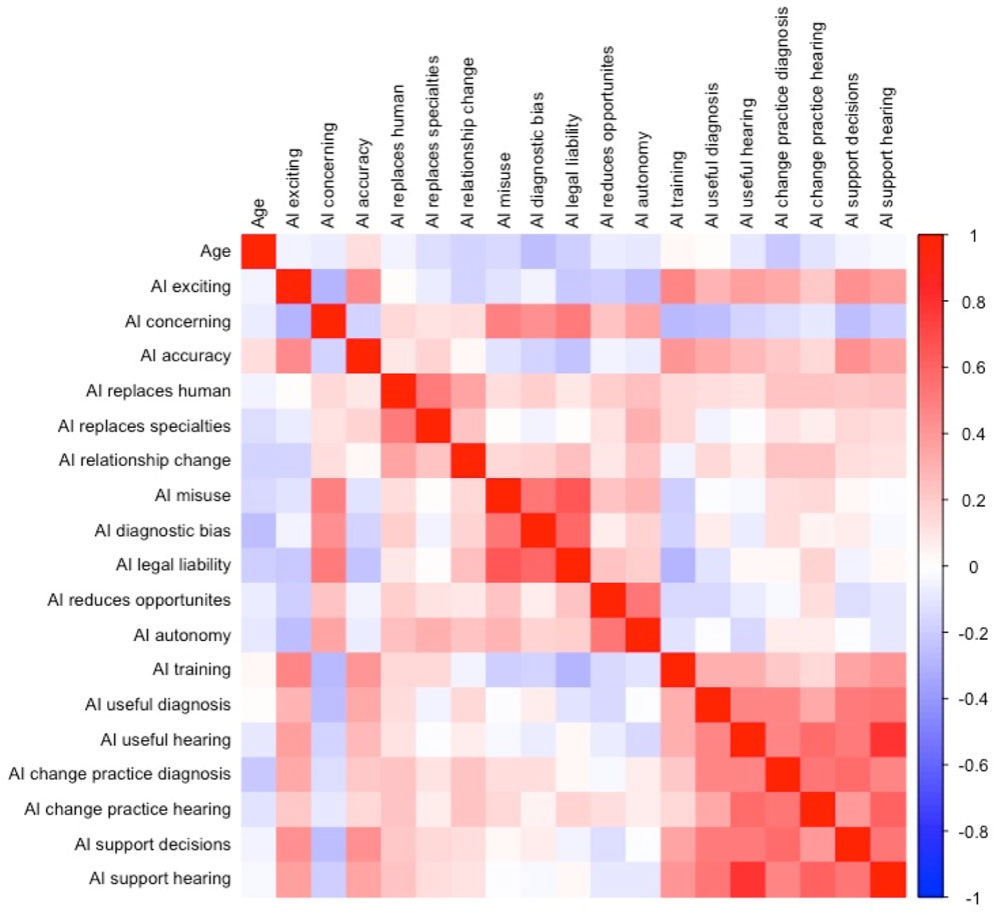
Spearman correlation matrix of age and responses to questions. Note: The Spearman correlation coefficient ranges from −1 to 1. A correlation of −1 indicates a perfect negative relationship, 0 indicates no linear relationship and 1 indicates a perfect positive relationship between two variables. The heatmap colour scale indicates the strength and direction of the correlations, where warmer colours (shades of red) represent positive correlations and cooler colours (shades of blue) represent negative correlations. The intensity of the colours reflects the magnitude of the correlation, with darker shades indicating stronger associations. See Table 1 for full question statements.

Participants expressed excitement towards AI technologies (mean: 4.14, median: 4) and showed openness to using AI for diagnosis and clinician decision support in their practice (mean: 3.98–4.02, median: 4). However, concerns were raised regarding potential impacts, including diagnostic bias (mean: 3.54, median: 4), data misuse (mean: 3.54, median: 4), legal liability (mean: 3.68, median: 4) and reduced learning opportunities for humans (mean: 3.29, median: 4). Participants agreed that AI training should be integrated into professional education and training programmes (mean: 4.17, median: 4).

Participants tended to disagree that AI technologies are more accurate than humans at diagnosis (mean: 2.82, median: 3), that AI would remove their autonomy (mean: 2.54, median: 2) or that AI would completely replace certain medical specialties (mean: 2.57, median: 2). However, participants leaned towards agreement that some human healthcare professionals would be replaced by AI in the future (mean: 3.31, median: 4).

Notably, there were no statistically significant differences in the attitudes expressed between the groups (ENT *vs* audiology *vs* general practitioners). The analysis, using the Kruskal–Wallis test, showed that the *p* values for all the comparisons were greater than 0.05, indicating that the observed variations in attitudes were likely due to chance rather than meaningful differences between the groups. Additionally, Spearman's correlation analysis revealed no significant correlation between the age of the participant and the opinions expressed about AI technologies in healthcare (Spearman's *r* < 0.3, *p* > 0.05) (Figure 3).

### Qualitative analysis of further comments

The final question of the survey asked participants to ‘please write any further comments you may have about AI technologies in hearing healthcare’.

Multiple readings of the participant responses was performed by one author (BO) to gain familiarity with the data and to identify patterns and concepts (known as ‘codes’ in qualitative research). The initial codes created were software and technology, concerns and ethics, potential benefits and applications, patient–clinician relationship, practical considerations and limitations, and uncertainties and balances.

After reflecting on the codes and interpreting them in the context of the research objectives, the codes were refined into five themes. In qualitative analysis, a theme is a recurring pattern or concept found in a dataset and is made up of a collection of codes. The ‘software and technology’ code was not included as a theme as it did not capture a distinct recurring pattern in the data. The themes and examples of statements were concerns and ethical considerations, potential benefits and applications of AI in hearing healthcare, the patient–clinician relationship and the human component, practical considerations and limitations, and uncertainties and a balanced perspective.

#### Concerns and ethical considerations

The comments provided by the respondents highlighted concerns regarding the perceived lack of diversity within the community of AI developers and many of the datasets the algorithms are trained on. This could potentially lead to harms and unintended consequences through bias and limitations in performance. This observation is significant as it prompts inquiries into the notions of fairness, equity and the possibility of AI technologies aggravating prevailing health disparities if they are not deployed in a responsible and equitable manner.

‘I think AI technology is in its infancy and this is both exciting and concerning as to its application in healthcare. In certain scenarios AI can be used to enhance healthcare but we need to consider the impacts of this new technology and potential harms.’

‘Largely based codes [sic] regression models written by white men.’

#### Potential benefits and applications of artificial intelligence in hearing healthcare

The respondents acknowledged the potential benefits of AI in hearing healthcare, particularly in the areas of diagnosis and analysis. They expressed the view that AI has the capacity to serve as a valuable tool during these phases of hearing healthcare delivery and considered it as an intriguing prospect for the future. The respondents also recognised the widespread influence of AI in various technical fields, highlighting its significance as a transformative force shaping the future of hearing healthcare and other industries.

‘Likely to be useful tools during diagnostic and analytic phases.’

‘Anything to aid our assessment and diagnosis of patients within a consultation would be an interesting prospect for the future.’

‘Very useful in helping to separate ear conditions that require urgent referrals from those that require routine referral to ENT.’

#### Patient–clinician relationship and human component

Respondents acknowledged the continued significance of the human component in the patient–clinician relationship, which emphasised the importance of empathy, and recognised that patients value interactions with human clinicians. They viewed AI as a supportive tool rather than a complete replacement for human clinicians. The respondents further acknowledged the influence of patient preferences and expectations. These insights contribute to our understanding of the complex dynamics between AI, healthcare professionals and patients.

‘The human component is still a very important part of patient–clinician relationship.’

‘I am interested in how AI can improve and help clinicians make diagnoses and treat patients.’

‘Patients want what they want … If they want to see someone, for example, they will change the answers until they get to see someone.’

#### Practical considerations and limitations

The respondents' perspectives reveal some practical considerations and limitations associated with the integration of AI in hearing healthcare. One important aspect highlighted was the need for clinicians to receive adequate training and access to necessary resources to effectively utilise AI technologies. They also emphasised the time pressures often faced in primary care settings, suggesting that the implementation of AI should be mindful of the demanding nature of these environments. In addition, concerns were raised about patients potentially finding it difficult to understand instructions if they had to use AI technologies. These insights underscore the importance of addressing practical barriers to ensure seamless integration of AI into hearing healthcare workflows while considering the unique context and dynamics of clinical practice.

‘Requires appropriate training and resources for clinicians.’

‘May be time-pressured in general practitioner settings.’

‘Patients can be very difficult to test and there are issues with understanding the instructions. Often you can tell by watching a patient carefully whether they have heard a sound or not. Tinnitus can make responses very variable, which can be allowed for by the clinician. Would AI be able to do this?’

‘Patients want what they want. They therefore have to tell the truth. If they are not happy with the outcome they just rerun the inquiry until they get the answer they want. If they want to see someone, for example, they will change the answers until they get to see someone.’

#### Uncertainties and balanced perspective

The participants' feedback regarding uncertainties and maintaining a balanced perspective regarding AI in hearing healthcare provided valuable insights. Participants acknowledged that AI technology is still in its early stages of development. There is a prevailing belief that changes in this field will occur gradually, with advancements happening once the technologies have matured sufficiently. While the current state of AI in hearing healthcare presents both excitement and concern, it highlights the need for careful exploration and evaluation of the applications of AI in hearing healthcare, keeping in mind the ethical, social and practical implications to maintain a balanced perspective.

‘Changes will happen slowly and only when technologies are mature.’

‘Do not feel hearing healthcare should be focus for AI.’

‘AI technology is in infancy and this is both exciting and concerning as to application in healthcare.’

‘The robots better not be taking my job. Those are MY audiometer dials to twiddle … not Chappie's!’

## Discussion

### Principal findings

The majority of the respondents were younger in age and rated themselves as ‘tech savvy’, believed that they understood the concept of AI and were aware of AI being used in healthcare. The participants in this study expressed excitement and openness towards AI in healthcare, highlighting the potential benefits during the diagnostic and analytic phases of hearing healthcare delivery. However, they also raised concerns about ethical considerations, including the potential for biases, data misuse, a lack of clarity about legal liability and the need to address the impacts and potential harms associated with AI technologies. Healthcare professionals consider fairness and equity in the development and deployment of AI as important factors in ensuring existing health inequalities are not widened.

- Clinician acceptance impacts technology adoption, yet little is known about the attitudes of UK hearing health professionals towards artificial intelligence (AI) in hearing healthcare
- A web-based survey was conducted with UK ENT specialists, audiologists and general practitioners to assess their attitudes to AI in hearing healthcare
- The findings highlight diagnostic quality, time efficiency and user-centred design as important considerations for AI adoption and implementation
- Understanding the sociotechnical context of the local healthcare system and the emotional element of attitudes to AI technologies is vital to avoid implementation failures
- Future studies should focus on understanding and addressing specific concerns and challenges within each speciality to ensure safe and responsible integration of AI technologies in hearing healthcare

While healthcare professionals providing hearing healthcare noted the potential advantages of using AI technologies, in their response to questions regarding the impact of AI technologies on the patient–clinician relationship they highlighted the need for AI technologies not to replace the human interpersonal aspect of the patient–clinician relationship and for patient preferences to remain central to hearing healthcare delivery. Participants also identified some practical considerations and limitations associated with the integration of AI in hearing healthcare. Adequate training and resources for healthcare professionals were emphasised as essential for the effective use of AI, while the time pressures often faced in primary care settings was recognised as a potential limitation to adoption.

Participants believed that changes in AI technologies would occur gradually as the technologies mature and a balanced perspective is required. There was scepticism about the utility of AI in certain interpersonal situations, underscoring the importance of careful consideration and evaluation when implementing AI technologies in hearing healthcare. These insights emphasise the need to address practical barriers and ensure successful integration of AI into hearing healthcare workflows while considering the unique dynamics of clinical practice.

It was interesting to note that there were no significant differences between the professions in their opinions about AI. This contrasts with attitudes in the 1970s, when general practitioners were at the forefront of the revolution in computerised health service provision while secondary care professionals were slow to adopt it.^21^ In addition, the age of the participants showed only weak correlations with agreement on the survey statements. This suggests that there may be broad agreement on how AI technology should be developed and deployed.

### Comparison with the literature

This is the first study investigating attitudes to AI amongst a range of healthcare professionals providing hearing healthcare. No studies have specifically investigated attitudes amongst ENT specialists or audiologists, highlighting the gap in the literature that this study fills.

The only previous survey investigating attitudes to AI amongst UK general practitioners was published by Blease *et al*. and included 1474 general practitioner respondents.^22^ It revealed that general practitioners expressed considerable optimism about the likelihood that clinical documentation would be fully automated soon, with the majority of participants (79 per cent) believing this would occur within 10 years. In the same study, respondents believed it unlikely that technology will ever be able to fully replace physicians when it comes to diagnosing patients (68 per cent) and delivering empathic care (94 per cent). Artificial intelligence technologies have significantly advanced since the Blease *et al*. paper was published, yet the present study also found that hearing health professionals did not believe that AI technologies would replace human clinicians entirely. In addition, this study found that hearing health professionals believed the human component of the patient–clinician relationship would not easily be replicated by AI technologies.

Two qualitative studies have been conducted with general practitioners exploring their opinions on AI in healthcare. In a follow up to their questionnaire, Blease *et al*. conducted a web-based survey of 720 UK general practitioners to gauge their opinions on the likelihood of future technology fully replacing general practitioners in performing 6 key primary care tasks and, if respondents considered replacement for a particular task likely, their estimate of how soon the technological capacity might emerge.^23^ The survey identified three major themes: the limitations of future technology, the potential benefits of future technology, and social and ethical concerns. The limitations included the belief that AI will not be able to replicate the human ability to show empathy. The perceived benefits were improved efficiency and a reduced burden on clinicians. Social and ethical concerns were varied and included the acceptability of AI to patients, the potential to cause harm and, tangentially, AI's potential impact on understaffing.

Buck *et al*. interviewed 18 general practitioners from Germany in 2020 to better understand general practitioners’ attitudes to AI-enabled systems.^24^ They found three determinants of their attitudes: concerns, expectations and minimum requirements. Concerns regarding AI-enabled systems included existential anxiety, the potential change in the physician–patient relationship, misuse of data and diagnostic bias. Some participants expressed fears of AI taking over their tasks and feeling replaceable. There were concerns about the impact of AI on the physician–patient relationship, including the potential loss of personal attention and standardised interactions.

Misuse of data and violation of privacy were also major concerns, with participants worried about data being intercepted and used to the disadvantage of patients. Diagnostic bias was another concern, as AI systems could influence decision-making and lead to incorrect diagnoses and overexpansion of treatment services.

General practitioners had positive expectations regarding the benefits of AI in terms of diagnostic quality, diagnostic efficiency and legal liability, but they also had concerns about AI's lack of human competencies and the potential increase in time expenditure. Environmental influences, such as changing working conditions, stakeholder opinions, media coverage and information technology infrastructure, also shaped general practitioners' attitudes. Individual characteristics, such as age and affinity with technology, influenced the participants’ perspectives.

The minimum requirements for AI-enabled systems, as identified from the interviews, included time efficiency, diagnostic quality, data security, economic viability, transparency and autonomy. General practitioners expect AI systems to be fast and easy to use, provide accurate diagnoses, ensure data privacy, be affordable, be transparent in their functioning and allow physicians to maintain their autonomy in decision-making. It is important to note that in some respects, clinicians’ autonomy in decision-making has been progressively limited over recent decades by clinical guidelines and targets (e.g. quality and outcomes framework indicators), so AI technologies used for decision-support would not be spoiling a ‘perfect picture’.

This study's findings share similar themes with those published in the literature, including the perceived benefits of AI in hearing healthcare, its limitations, and social and ethical considerations. However, in contrast to the findings of Buck *et al*.,^24^ we did not find a significant association between age and the attitudes expressed by general practitioners.

One common theme across all studies was the belief that AI technologies could not replicate the human clinician's ability to interact empathetically with their patients. However, recent advancements in large language models, such as ChatGPT^TM^, have demonstrated the ability of AI to generate quality and empathetic responses to patient questions. Evaluators in one study preferred the chatbot's responses to physicians in 78.6 per cent of the 585 evaluations (95 per cent confidence interval, 75.0–81.8 per cent) and rated the chatbot's responses as significantly more empathetic than physician responses (*t* = 18.9, *p* < 0.001).^25^ Although further research is required to explore this application of AI in healthcare, it has clear implications for the future of the patient–clinician relationship.

These findings highlight the importance of addressing the emotional component of attitudes to AI technologies, and have identified diagnostic quality and time efficiency as crucial factors for healthcare professionals to consider in adopting AI systems. User-centred design and proactive promotion of AI-enabled systems are essential for the successful integration of AI systems into everyday use in hearing healthcare. Ignoring the surrounding sociotechnical context of the complex circumstances of the healthcare system could lead to implementation failure (e.g. unintended effects, non-adoption or abandonment).^26,27^

### Strengths and limitations

This study has some limitations inherent with cross-sectional surveys. Firstly, the sample size was small, with a relatively young cohort of respondents and may not represent the views of the broader population of professionals working in the UK. There could also be inherent bias in participants' willingness to participate, introducing potential biases in the attitudes expressed in this survey. Furthermore, the qualitative data obtained were only volunteered by a proportion of the respondents, and it was not possible to probe the participants' responses because of the limitations of the online survey. Further qualitative work with ENT specialists and audiologists would address this knowledge gap.

## Conclusions

This mixed-methods analysis provides insights into the understanding, experience and attitudes of audiologists, ENT specialists and general practitioners in the UK towards AI technologies in hearing health. The findings highlight the need for appropriate training and resources, careful consideration of ethical implications, a patient- and user-centred approach, and a balanced perspective on the potential benefits and limitations of AI for clinical decision support. Future research should involve the co-design and co-creation of solutions to the concerns highlighted by hearing healthcare professionals in this study.

## Supporting information

Oremule et al. supplementary material 1 Oremule et al. supplementary material

Oremule et al. supplementary material 2 Oremule et al. supplementary material

Oremule et al. supplementary material 3 Oremule et al. supplementary material

## Supplementary material

The supplementary material for this article can be found at https://doi.org/10.1017/S0022215124000550.
