# Supplementary material for: Understanding, experience and attitudes towards artificial intelligence technologies for clinical decision support in hearing health: a mixed-methods survey of healthcare professionals in the UK
Source: J Laryngol Otol. 2024 Apr 18;138(9):928–35. doi: 10.1017/S0022215124000550 (PMC11518668; doi:10.1017/S0022215124000550)
Supplement: Oremule et al. supplementary material 1 — Oremule et al. supplementary material [file S0022215124000550sup001.docx]

**Included studies in scoping review**

1. Adeeb, N., Hattab, T., Savardekar, A., Jumah, F., Griessenauer, C. J., Musmar, B., Adeeb, A., Trosclair, K., & Guthikonda, B. (2021). Venous Thromboembolism Prophylaxis in Elective Neurosurgery: A Survey of Board-Certified Neurosurgeons in the United States and Updated Literature Review. World Neurosurgery, 150, e631–e638.
2. Ahmed, Z., Bhinder, K. K., Tariq, A., Tahir, M. J., Mehmood, Q., Tabassum, M. S., Malik, M., Aslam, S., Asghar, M. S., & Yousaf, Z. (2022). Knowledge, attitude, and practice of artificial intelligence among doctors and medical students in Pakistan: A cross-sectional online survey. Annals of Medicine and Surgery, 76.
3. Blease, C., Bernstein, M. H., Gaab, J., Kaptchuk, T. J., Kossowsky, J., Mandl, K. D., Davis, R. B., & DesRoches, C. M. (2018). Computerization and the future of primary care: A survey of general practitioners in the UK. PLoS ONE, 13(12).
4. Blease, C., Kaptchuk, T. J., Bernstein, M. H., Mandl, K. D., Halamka, J. D., & Desroches, C. M. (2019). Artificial intelligence and the future of primary care: exploratory qualitative study of UK general practitioners’ views. Journal of Medical Internet Research, 21(3).
5. Buck, C., Doctor, E., Hennrich, J., Jöhnk, J., & Eymann, T. (2022). General Practitioners’ Attitudes Toward Artificial Intelligence–Enabled Systems: Interview Study. Journal of Medical Internet Research, 24(1).
6. Galli, B., & Chong, J. (2019). Artificial intelligence in radiology: who’s afraid of the big bad wolf? European Radiology, 29(4), 1637–1639.
7. Ho, S., Doig, G. S., & Ly, A. (2022). Attitudes of optometrists towards artificial intelligence for the diagnosis of retinal disease: A cross-sectional mail-out survey. Ophthalmic and Physiological Optics, 42(6), 1170–1179.
8. Holzner, D., Apfelbacher, T., Rödle, W., Schüttler, C., Prokosch, H. U., Mikolajczyk, R., Negash, S., Kartschmit, N., Manuilova, I., Buch, C., Gundlack, J., & Christoph, J. (2022). Attitudes and Acceptance Towards Artificial Intelligence in Medical Care. Studies in Health Technology and Informatics, 294, 68–72.
9. Palmisciano, P., Jamjoom, A. A. B., Taylor, D., Stoyanov, D., & Marcus, H. J. (2020). Attitudes of Patients and Their Relatives Toward Artificial Intelligence in Neurosurgery. World Neurosurgery, 138, e627–e633.
10. Pinto dos Santos, D., Giese, D., Brodehl, S., Chon, S. H., Staab, W., Kleinert, R., Maintz, D., & Baeßler, B. (2019). Medical students’ attitude towards artificial intelligence: a multicentre survey. European Radiology, 29(4), 1640–1646.
11. Polesie, S., Gillstedt, M., Kittler, H., Lallas, A., Tschandl, P., Zalaudek, I., & Paoli, J. (2020). Attitudes towards artificial intelligence within dermatology: an international online survey. In British Journal of Dermatology (Vol. 183, Issue 1, pp. 159–161).
12. Scott, I. A., Carter, S. M., & Coiera, E. (2021). Exploring stakeholder attitudes towards AI in clinical practice. In BMJ Health and Care Informatics (Vol. 28, Issue 1).
13. van Hoek, J., Huber, A., Leichtle, A., Härmä, K., Hilt, D., von Tengg-Kobligk, H., Heverhagen, J., & Poellinger, A. (2019). A survey on the future of radiology among radiologists, medical students and surgeons: Students and surgeons tend to be more skeptical about artificial intelligence and radiologists may fear that other disciplines take over. European Journal of Radiology, 121.
14. Wood, E. A., Ange, B. L., & Miller, D. D. (2021). Are We Ready to Integrate Artificial Intelligence Literacy into Medical School Curriculum: Students and Faculty Survey. Journal of Medical Education and Curricular Development, 8, 238212052110240.
15. Young, A. T., Amara, D., Bhattacharya, A., & Wei, M. L. (2021). Patient and general public attitudes towards clinical artificial intelligence: a mixed methods systematic review. In The Lancet Digital Health (Vol. 3, Issue 9, pp. e599–e611).
16. Zheng, B., Wu, M. nian, Zhu, S. jun, Zhou, H. xia, Hao, X. lan, Fei, F. qin, Jia, Y., Wu, J., Yang, W. hua, & Pan, X. ping. (2021). Attitudes of medical workers in China toward artificial intelligence in ophthalmology: a comparative survey. BMC Health Services Research, 21(1).
