# Supplementary material for: Understanding, experience and attitudes towards artificial intelligence technologies for clinical decision support in hearing health: a mixed-methods survey of healthcare professionals in the UK
Source: J Laryngol Otol. 2024 Apr 18;138(9):928–35. doi: 10.1017/S0022215124000550 (PMC11518668; doi:10.1017/S0022215124000550)
Supplement: Oremule et al. supplementary material 2 — Oremule et al. supplementary material [file S0022215124000550sup002.docx]

**
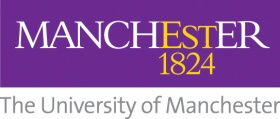
**

**What are healthcare professionals’ attitudes to artificial intelligence in hearing health?**

*Long title: Understanding, experience, and attitudes towards Artificial Intelligence technologies for clinical decision support in hearing health: survey of healthcare professionals in the UK*

**Questionnaire**

| Demographic data | | | | | | |
| --- | --- | --- | --- | --- | --- | --- |
| Age at recruitment | - [Free text box] | | | | | |
| Sex | - Male - Female - Prefer not to say | | | | | |
| Occupation | - Audiologist - ENT - General Practitioner | | | | | |
| Grade | **Audiologist**   - Band 4 - Band 5 - Band 6 - Band 7 - Band 8a - Band 8b - Band 8c - Band 8d - Non AfC band   **ENT specialist**   - Trainee ST3-5 (or equivalent experience) - Trainee ST6-8 (or equivalent experience) - Consultant - Associate Specialist   **General Practitioner**   - GPST1 - GPST2 - GPST3 - GP < 5 years post-qualification - GP 5-10 years post-qualification - GP > 10 years post-qualification | | | | | |
| Region of practice | - East of England - East Midlands - West Midlands - London - North East - North West - Yorkshire - South-East - South-West   **Northern Ireland**   - County Antrim - County Armargh - County Down - County Fermanagh - County Londonderry - County Tyrone   **Scotland**   - Central Scotland - Glasgow - Highlands and Islands - Lothian - Mid-Scotland and Fife - North East Scotland - South Scotland - West Scotland   **Wales**   - North Wales - Mid Wales - South West Wales - South East Wales | | | | | |
| Qualifications | **Do you have any formal training in artificial intelligence in healthcare?**   - Yes - No   **If yes, please state level of training e.g. certificate, diploma, degree or other.**  [Free text] | | | | | |
| Your basic understanding and awareness of AI technologies | | | | | | |
| Are you confident using everyday technology, such as computers and smartphones? | - Yes - No - Somewhat - Unsure | | | | | |
| Are you familiar with the concept of AI? | - Yes - No - Somewhat - Unsure | | | | | |
| Are you familiar with uses for AI? | - Yes - No - Somewhat - Unsure | | | | | |
| Are you aware of AI being used in the medical field? | - Yes - No - Somewhat - Unsure | | | | | |
| To date where has most of your knowledge of AI technologies in healthcare come from? (Tick all that apply) | - Media/social media - Friends and family - Colleagues - Professional colleges and organisations - Published medical literature - Other [free text] | | | | | |
| Have you used any AI technologies in your practice? | - Yes - No - Unsure   If yes, please list examples - [free text] | | | | | |
| Your general attitudes to AI technologies in healthcare  For each of the statements below, please indicate your level of agreement from "strongly disagree" to "strongly agree" | | | | | | |
|  | Strongly disagree  1 | Disagree  2 | Neither agree nor disagree  3 | Agree  4 | | Strongly agree  5 |
| New AI technologies in healthcare are exciting |  |  |  |  | |  |
| New AI technologies in healthcare concern me |  |  |  |  | |  |
| In general AI technologies are more accurate than healthcare professionals at diagnosis |  |  |  |  | |  |
| Some human healthcare professionals will be replaced by AI in the foreseeable future |  |  |  |  | |  |
| AI will replace some medical specialties in the near future |  |  |  |  | |  |
| AI will change the relationship between members of my profession and their patients |  |  |  |  | |  |
| I have concerns about misuse of data with AI technologies in healthcare |  |  |  |  | |  |
| I have concerns about diagnostic bias in AI technologies in healthcare |  |  |  |  | |  |
| I have concerns about legal liability when using AI technologies in healthcare |  |  |  |  | |  |
| Using AI technologies in healthcare may reduce human physicians' learning opportunities and knowledge |  |  |  |  | |  |
| AI will remove my autonomy in making medical diagnoses |  |  |  |  | |  |
| AI in healthcare should be part of our professional education and training |  |  |  |  | |  |
| If your medical judgement and an AI algorithm’s differ, which would you likely follow? | - My judgement - The AI algorithm - Unsure | | | | | |
| Interest in AI technologies for clinical decision support in ear and hearing health | | | | | | |
|  | Strongly disagree  1 | Disagree  2 | Neither agree nor disagree  3 | Agree  4 | | Strongly agree  5 |
| AI software that could help with diagnosing ear conditions would be useful in my practice |  |  |  |  |  | |
| AI software that could measure hearing thresholds would be useful in my practice |  |  |  |  |  | |
| AI software that could help with diagnosing ear conditions would change my practice |  |  |  |  |  | |
| AI software that could help with measuring hearing thresholds would change my practice |  |  |  |  |  | |
| Would you like to use an AI software/programme that can be helpful in diagnosing ear conditions? | - Yes - No - Unsure | | | | | |
| Would you like to use an AI software/programme that can assist with measuring hearing thresholds if you knew the technology had good sensitivity and specificity? | - Yes - No - Unsure | | | | | |
| If you were able to take a video or image of the tympanic membrane during an ear and hearing assessment for an AI algorithm to analyse immediately, what would be your preferred device to use? | - Video-otoscope - Smartphone - Endoscope - Pen-shaped device - I would not be interested in such a device - Other (please state) [Free text] | | | | | |
| What would be your preferred interface for an AI clinical decision support application? | - Dedicated software on your computer - Integrated smartphone application - Online / web-based software - Addition to existing electronic patient record / business software - Dedicated device - Other [free text] | | | | | |
| Please write any further comments you may have about AI technologies in hearing healthcare | | | | | | |
| [Free text box] | | | | | | |

This concludes the questionnaire.

Thank you for taking part.
