# Supplementary material for: Understanding, experience and attitudes towards artificial intelligence technologies for clinical decision support in hearing health: a mixed-methods survey of healthcare professionals in the UK
Source: J Laryngol Otol. 2024 Apr 18;138(9):928–35. doi: 10.1017/S0022215124000550 (PMC11518668; doi:10.1017/S0022215124000550)

**
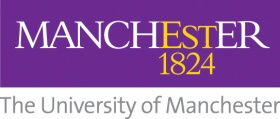
**

**What are healthcare professionals’ attitudes to artificial intelligence in hearing health?**

**Study invitation**

Dear colleague,

You have been invited to participate in a research project investigating healthcare professionals' attitudes towards artificial intelligence (AI) in ear and hearing health. As a practicing GP, audiologist, ENT specialist, ENT SAS grade doctor, or Doctor in Training (GPST or ENT ST) in these fields, your experiences are crucial in understanding the factors that affect perception of AI in hearing health. This research is funded by Health Education England and will contribute towards the award of a doctoral research degree.

Please follow this link to take a survey ([Take the Survey](https://www.qualtrics.manchester.ac.uk/jfe/preview/previewId/945d3028-0391-47f2-8013-93dcd8f65e79/SV_d1rwhDLBLJsHg8e?Q_CHL=preview)), which should take around 10-12 minutes to complete. You will only be required to participate only **once** in the survey cycle before it closes on 30th April 2023. 

The [Participant Information Sheet](https://www.qualtrics.manchester.ac.uk/CP/File.php?F=F_eVA4c1V8tDtm3rM) contains more detailed information about the study, details how you were selected and covers confidentiality safeguards.

Your participation is valued and confidential. 

Please do not hesitate to contact me if you have any questions at: [babatunde.oremule@postgrad.manchester.ac.uk](mailto:babatunde.oremule@postgrad.manchester.ac.uk)

Kind regards,

Tunde

Mr Babatunde Oremulé

Specialty Registrar in ENT, Head & Neck Surgery, Royal Manchester Children's Hospital

PhD Candidate, Division of Infection, Immunity and Respiratory Medicine, School of Biological Sciences, Faculty of Biology, Medicine and Health, University of Manchester.

**Follow this link to the Survey:**
[Take the Survey](https://www.qualtrics.manchester.ac.uk/jfe/preview/previewId/945d3028-0391-47f2-8013-93dcd8f65e79/SV_d1rwhDLBLJsHg8e?Q_CHL=preview)

Or copy and paste the URL below into your Internet browser:
<https://www.qualtrics.manchester.ac.uk/jfe/preview/previewId/945d3028-0391-47f2-8013-93dcd8f65e79/SV_d1rwhDLBLJsHg8e?Q_CHL=preview>

**Scan the QR code to the Survey:**

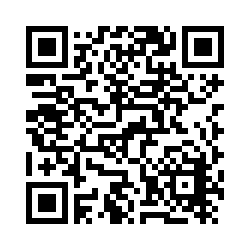

Supplement: Oremule et al. supplementary material 3 — Oremule et al. supplementary material [file S0022215124000550sup003.docx]
